# Effectiveness of Biofunctionalization of Titanium Surfaces with Phosphonic Acid

**DOI:** 10.3390/biomedicines9111663

**Published:** 2021-11-11

**Authors:** Ainhoa Aresti, Javier Aragoneses, Nansi López-Valverde, Ana Suárez, Juan Manuel Aragoneses

**Affiliations:** 1 Department of Medicine and Medical Specialties, Faculty of Medicine and Health Sciences, Universidad Alcalá de Henares, 28871 Madrid, Spain; aarestiallende@hotmail.com (A.A.); javias511@gmail.com (J.A.); 2 Department of Surgery, Instituto de Investigación Biomédica de Salamanca (IBSAL), University of Salamanca, 37007 Salamanca, Spain; nlovalher@usal.es; 3 Department of Preclinical Dentistry, School of Biomedical Sciences, Universidad Europea de Madrid, 28670 Madrid, Spain; 4 Dean of the Faculty of Dentistry, Universidad Alfonso X El Sabio, 28691 Madrid, Spain; jmaragoneses@gmail.com

**Keywords:** surface biofunctionalization, phosphonic acid, surface characteristics, scanning electron microscopy, oral implantology, fibroblasts, stem cells

## Abstract

Surface functionalization of dental implant surfaces has been a developing field in biomaterial research. This study aimed to obtain self-assembled monolayers (SAMs) using carboxyethylphosphonic acid on the surface of titanium (Ti) screws, and assessed the surface characteristics, biomechanical, and cellular behavior on the obtained specimens. This study had three groups, i.e., a control (untreated screws), a test group treated with phosphonic acid, and a third group with treated acid and bone morphogenetic protein (BMP-2) for in vitro analysis of cell lines. The assessed parameters included surface wettability, surface characteristics using scanning electron microscopy (SEM), protein immobilization, and cellular behavior of fibroblasts and mesenchymal stem cells of adipose tissue (MSCat cells). For surface wettability, a Welch test was performed to compare the contact angles between control (67 ± 1.83) and test (18.84 ± 0.72) groups, and a difference was observed in the mean measurements, but was not statistically significant. The SEM analysis showed significant surface roughness on the test screws and the cellular behavior of fibroblasts, and MSCat cells were significantly improved in this group, with fibroblasts having a polygonal shape with numerous vesicles and MSCat cells stable and uniformly coating the test Ti surface. Surface biofunctionalization of Ti surfaces with phosphonic acid showed promising results in this study, but remains to be clinically validated for its applications.

## 1. Introduction

Dental implants have been considered as an excellent fixed treatment option for the restoration of areas with a missing tooth/teeth [1]. Even though the TiO_2_ layer provides improved corrosion resistance and excellent physical load to the dental implants, it takes around 3–6 months for the implant to become biologically active and get attached to the surrounding bone (osseointegration) [2]. For improving the biological activity of Ti dental implants, various surface modification techniques to alter surface roughness, topography, chemistry, and electrical charge have been researched [3].

The primary methods of surface modification involve alteration in the topography. There are also processes where biomimetic and biologically active substances have been added to the dental implant surface for improving its biologic characteristics and potentiate osseointegration [4,5,6]. There are two conflicting properties involved in these biofunctional properties: they include enhancement or inhibition of protein adsorption/cell adhesion [7].

An attractive approach to modify the interfacial properties of Ti and its alloys is self-assembled monolayers (SAMs). They allow surface control and are easy to manipulate, therefore, bioactivity and biocompatibility of the implant can be achieved at a low cost. These types of layers have given rise to numerous studies [8,9,10,11,12].

Self-assembled monolayers (SAMs) for dental implants have been proposed as a processing approach for the modification of Ti surfaces at the nanoscale level. SAMs are formed by the immersion of substrate into an active surfactant solution in an appropriate solvent (organic or aqueous), or by methods such as vapor deposition or aerosol spraying. Immersion remains the easiest and cheaper method for its application in substrates with complex geometrics [13,14].

Liu et al. [15] explored a strategy to enhance Ti osseointegration through the formation of alkyl-based SAM in a Ti foil using end groups such as carboxyl, hydroxyl, vinyl, and phosphate. It was reported that hydroxyapatite (HA) coating was obtained in carboxyl and phosphate end groups when Ti foil was immersed in a solution that contained ions with 1.5 times greater concentration than simulated body fluid (SBF). Another study by Liu et al. [16] investigated the optimal functional end group for the formation of biomimetic HA on Ti surfaces. The study concluded that the carboxylic acid end group provided the optimal SAM for the formation of HA, and it was suggested that the affinity of carboxyl groups to CaP played a crucial role in the surface crystallization of HA. Also, the alkyl chain length of phosphonic acid also plays an important role for the formation of crystalline HA [16].

Even though there are studies that show that carboxyl groups in SAMs are capable of inducing HA crystallization, there is a dearth in evidence on cell proliferation/adhesion properties, biocompatibility, and osteogenic potential of the treated surfaces. The hypothesis for this in vitro study was based on the application of carboxyethyl phosphonic acid on the Ti implant surface to obtain a monolayer of carboxyl groups that may give rise to an increase of wettability, and a hydrophilic surface capable of generating stable links, and which might serve as an intermediate layer for protein combination. The present study aimed to analyze the physico-chemical and biological properties of Ti surfaces treated with carboxyethylphosphonic acid that form SAMs by the immersion method.

## 2. Materials and Methods

In this in vitro study, three study groups were utilized to understand the differences between untreated Ti screws, the Ti screw surface treated with carboxylphosphonic acid resulting in the formation of SAMs, and the third group treated with acid and bone morphogenetic protein (BMP-2).

- Untreated Ti screws;
- Ti screws treated with carboxylphosphonic acid using the immersion method;
- Ti screws treated with acid + BMP-2- (additional group during cellular behavior assay).

### 2.1. Surface Modification: Carboxyethylphosphonic Acid Treatment

The samples used in this study were Ti alloy (titanium (90%), aluminum (6%), vanadium (4%) (*n* = 10)) which underwent a process of immersion for a period of 24 h at 76 °C in a mixture made with 50 mL of tetrahydrofuran (THF) and 55 mg of carboxyethylphosphonic acid. The preparation was performed in a three-neck flask and, to keep the constant, the THF in the required linear reflux at 5 °C. After 24 h, the samples were removed, and the next step was to activate the carboxyethylphosphonic acid. For the activation, 3-dimethylaminopropyl carboxylamide (EDC or EDAC) and NHS (N-hydroxysulfamide) compounds were used.

The TiO_2_ surface has many OH groups on the surface with an internal Ti-O-Ti structure. The application of a phosphonate will remove H_2_O upon heating, leaving a molecule with an arrangement of carboxyl groups (COOH) on the surface.

**Figure 1:**
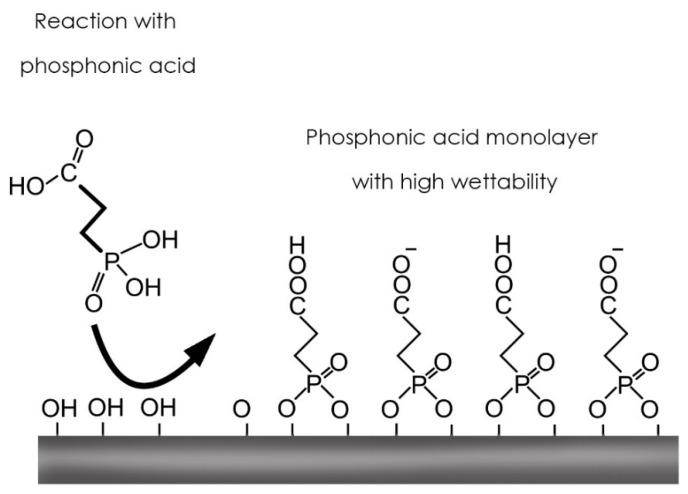
Scheme of the molecular reaction of carboxyethylphosphonic acid on the titanium surface. The black arrow indicates when carboxyethylphosphonic acid contacts and bonds to the OH groups of TiO_2_.

H_2_O leaves a molecule with an arrangement of carboxyl groups (COOH) on the surface, capable of generating stable bonds with protein molecules (Figure 1).

**Figure 2:**
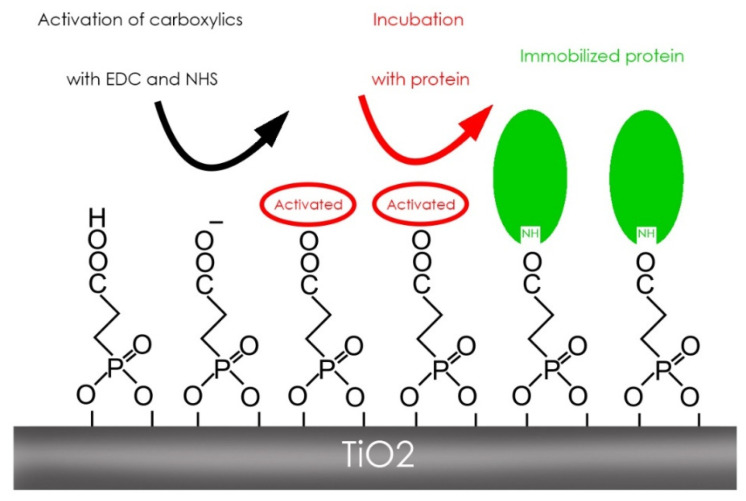
Scheme of the binding process from activation of carboxyl groups, incubation with the protein, and immobilization of the protein.

The EDC was used to activate carboxyl groups and couple amines, and it reacts with the carboxyl groups to form an intermediate of O-acylisourea amine reagent. In case the intermediate does not link with a carboxyl group, it will hydrolyze and regenerate a carboxyl group—NHS was utilized for this phase. In the presence of NHS, EDC can be used to convert carboxyl groups to sulfo-NHS ester reactive aine. The activation of carboxyethylphosphonic acid was completed using NHS and EDC compounds, where Ti samples were immersed in a preparation of EDC + NHS for 15 min at room temperature. The preparation is performed with 5 mL of water mixed with 175 mL of EDC plus 54 mg NHS—it reduced the pH of the preparation with hydrochloric acid to 7 [17] (Figure 2).

#### 2.1.1. Wettability

**Figure 3:**
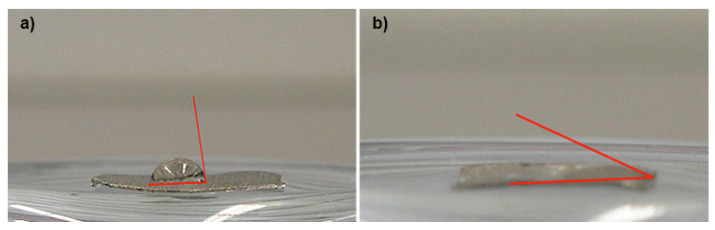
Wettability tests. (**a**) On the titanium surface of the control group. (**b**) On the surface treated with carboxyethylphosphonic acid.

The surface wettability test was carried out by placing a drop of H_2_O on 10 samples of Ti alloys treated with carboxyethylphosphonic acid, and 10 samples of untreated Ti (control group). A photograph of the system was taken, and contact angle measurement was performed by means of a computer program (Imane Pool-ITZIP). Scanning electron microscopy analysis was performed to evaluate the surface spectra in both the control and test groups (Figure 3).

#### 2.1.2. Surface Texture Analysis

The surface spectra of both acid-treated and untreated screws were examined using environmental SEM equipped with an X-ray emission probe that provided the necessary characteristics for measuring the surface chemical composition of different biomaterials. The SEM analysis was performed at low magnification (50×) on the cranial side of the screws between the study groups. It is vital to dehydrate and process the specimens for the observation under SEM. Firstly, the specimens were dehydrated by immersing them in a graded series of ethanol from 30% to absolute ethanol. Once dehydrated, the samples were taken to a critical point of total dehydration. A Polaron 3000 (Carl Zeiss Meditec, Dublin, CA, USA) was used to perform this function using high vacuum.

The parts were then mounted on aluminum supports (stub), and sputter coated. The process consisted of depositing a thin layer of gold/palladium on the surface of the samples to increase their conductivity. A Zeiss metallizer was utilized for this process, and once metallized, the samples were prepared for observation by SEM (Zeiss DMS 950). The metallization may not have been necessary for the observation of the screw surface since Ti is a metal, however, by means of gold/palladium coating, we ensure perfect conductivity and additional functionality [18,19,20]—However, dehydration is mandatory. This is because the water molecules that may have been retained during the treatment with an aqueous solution need to be eliminated prior to SEM observation.

#### 2.1.3. Protein Immobilization

The immobilization of the model protein used (glucose oxidase at the Ti interface) was evaluated by fluorescence of the sample, thus, allowing the corroboration of the protein binding. The efficiency of the reaction and the presence of protein was measured using a fluorescein-label (NHS-Fluorescein). This molecule can react with the amine groups of the proteins, thereby producing a fluorescent group at 530 nm. The appearance of this probe can be correlated to the presence of the immobilized protein on the Ti surface.

Once the immobilization of protein on the surface of the modified Ti has been checked, in vitro studies were carried out to assess the cell behavior of two cell lines: fibroblasts and stem cells from adipose tissue (MSCat). In vivo studies were carried out to analyze the biocompatibility.

### 2.2. Cellular Behaviour of Fibroblasts and MSCat Cells

#### 2.2.1. Preparation of the Cultivation Medium

- Amniomax (Life Technologies, Inc., Gibco/BRL, Gaithersburg, MD, USA): According to the manufacturer’s package.
- Medium 199: 9.82 g of M-199 (Life Technologies, Inc., Gibco/BRL, Gaithersburg, MD, USA) was diluted in 1 L of ultrapure water, followed by the addition of sodium bicarbonate, and diluted through a 0.22 µm porous filter (Millipore Ref.4480). Once aliquoted into 100 mL fractions, the culture medium was supplemented with: 20% fetal bovine serum (FCS) (Life Technologies, Inc., Gibco/BRL, Gaithersburg, MD, USA); 1.2 mL of penicillin/streptomycin; 2 Mm/L hydroxyl-ethyl-piperazine-ethane-sulfonic acid (HEPES buffer) (Life Technologies, Inc., Gibco/BRL, Gaithersburg, MD, USA); 2 Mm/L-Glutamine (Gibco BRL); 20 µg/mL endothelial cell growth factor (ECGF) (Sigma Aldrich, San Luis, MO, USA); and 90µg/mL heparin sodium (Roche, Basel, Switzerland).
- Minimum Essentials Medium Eagle-MEM (Sigma Aldrich, San Luis, MO, USA). It was reconstituted with 15% fetal bovine serum (Cultek), 1% L-glutamine (Sigma-Aldrich, San Luis, MO, USA), and 1% antibiotics (penicillin and streptomycin) (Life Technologies, Inc., Gibco/BRL, Gaithersburg, MD, USA) in 1 L of ultrapure water—2.2 g of sodium bicarbonate was added and filtered through a 0.22 µm porous filter (Millipore Ref.4480). The medium was then aliquoted into 100 mL glass bottles, and supplemented with 0.05 lm Amphotericin B (Fungizone, Applied Biological Materials, Richmond, BC, Canada) and 1.2 mL penicillin (10,000 IU/mL)/Streptomycin (10,000 µg/mL) (Life Technologies, Inc., Gibco/BRL, Gaithersburg, MD, USA).

#### 2.2.2. Proteolytic Enzyme Preparation

Two proteolytic enzymes (collagenase and trypsin) were used. Collagenase was used to obtain cells from the different tissues, and trypsin was used to perform the different subcultures from cells in primary culture.

- Collagenase. 100 mg of collagenase type I CLS (Worthington Biochemical Corporation, Lakewood, New Jersey, USA) was reconstituted in 100 mL of MEM, and 1.5 mL of calcium chloride was added. One the reconstitution was performed, it was sterilized by filters of 0.22 µm (Millipore Ref.4480), and stored at −20 °C in 10 mL aliquots until usage.
- Trypsin. 10 mL of Hanks Balanced Buffer Saline Solution (HBSS) (Life Technologies, Inc., Gibco/BRL, Gaithersburg, MD, USA) was added to 90 mL of sterile distilled water, mixed well, and then, 10 mL of this solution was added to another 10 mL of trypsin EDTA 10X (Life Technologies, Inc., Gibco/BRL, Gaithersburg, MD, USA) to obtain trypsin-EDTA 1X.

#### 2.2.3. Cell Procurement and Cell Expansion in Culture

- Mesenchymal Stem Cells from Adipose Tissue (MSCat). For the extraction of stem cells from rat adipose tissue (MSCat), a sample of subcutaneous adipose tissue was procured from the inguinal area of the rat, and preserved in MEM medium until processing (no more than 24 h). The tissue processing was performed under sterile conditions using sterile materials and a laminar flow cabinet. At first, the tissue was washed with MEM medium, then placed in a Falcon tube with 10 mL of 0.1% collagenase type I, and incubated for 30 min at 37 °C in the bath with maximum agitation.

Following incubation, the suspension was passed through a 100 µm porosity filter in order to separate the adipose tissue fragments that had not been digested. The filtered portion was placed in a clean Falcon tube, and centrifuged at 1050 rpm for 7 min at room temperature. The supernatant was removed, and the sediment was re-suspended in MEM medium. The recovered tissue was washed again with MEM medium, and, using scalpel blades (No. 21), the tissue was cut into smaller explants that would get introduced in a Falcon tube with 10 mL of trypsin, and incubated for 30 min at 37 °C in the bath with maximum agitation.

Meanwhile, the sterile material used to filter the product of the enzymatic digestion was prepared: the plunger was removed from a 50 mL syringe, a sterile gauze was introduced into it with the help of forceps, and a Falcon tube with 10 mL of MEM was prepared. After 30 min, the digestion product was poured into the syringe, the plunger was then placed and filtered through the gauze, dropping the filtered product into the Falcon tube with MEM. The cell suspension obtained was centrifuged at 1050 rpm for 10 min at room temperature. The supernatant was removed, and the pellet was re-suspended in MEM medium for washing. This step was repeated twice to wash the sample well and remove excess fat.

During the last wash, the collagenase enzyme prepared at the beginning was used on the extract. It was re-suspended and placed on ice for 5 min. After this process, the cell suspension will get separated from the lipid component following the digestion of the adipocytes, and the lipid layer will remain on the top. With the help of a glass Pasteur pipette, the cell suspension was removed carefully without disturbing the lipid layer. The suspension was then centrifuged at 1050 rpm for 10 min at room temperature. The supernatant was removed, and the sediment (adipose tissue stem cells) was re-suspended in 3–6 mL of Amniomax medium, and transferred to 1–2 culture flasks of 25 cm^2^, which were stored with the cap slightly open in an incubator with an atmosphere of 5% CO_2_, 37 °C, and a humid environment.

- Fibroblasts

The procedure used to procure fibroblasts consisted of introducing the skin biopsy in a small Petri dish with a small amount of MEM, so that the tissue did not undergo dehydration during processing. The dermis was isolated using forceps and scissiors. Once the dermis was isolated, the tissue was cut using scalpel blades until small fragments (explants) of 0.5–1 mm^2^ were obtained. Subsequently, 1mL of Amniomax culture medium was added in a 25 cm^2^ culture flask, moving it gently so that the medium diffused over the entire expansion surface.

Using a glass Pasteur pipette, the explants were collected and then stuck to the expansion surface of the culture flask without sliding to the base of the flask. The culture flask was introduced into the CO_2_ oven at 37 °C, placed in a vertical position with the stopper slightly open to allow gas exchange in the incubator environment.

After a few hours, the flask was removed from the oven, previously closing the stopper—in the hood, 1 mL culture medium was added while taking special care to not detach the explants. The flask should be handled gently, and the volume of the culture medium should not exceed 2.5 mL to avoid detachment and floating of the explants. The flask was then placed back inside the CO_2_ oven with the stopper slightly open, this time in a horizontal position.

### 2.3. Proliferation Study

Cell cultures were performed to see the cellular behavior of fibroblasts and MSCats in an incubation period of 24 h in Amiomax medium. After seeding, all the prostheses obtained were fixed with 3% glutaraldehyde for 2–4 h, and then washed with MILLONIG buffer. Once fixed, the pieces were dehydrated using increasing series of acetone for 10 min per series. Subsequently, the dehydrated pieces were mounted in filter paper cases in order to subject them to a critical point in a Polaron E-3000 with CO_2_. Once completely dried, the pieces were mounted on steel plates (ANAME), and metallized with gold-palladium in a POLARON metallizer. In this way, the samples were prepared for observation by scanning electron microscopy in a DSM-950 microscope (ZEISS).

The samples, fixed in Bouin’s liquid, were dehydrated and embedded in kerosene, and then, blocks were cut with a MICROM HM 325 microtome into 5 μm thick sections. After this procedure, immunohistochemical techniques were performed.

### 2.4. Migration Study

A parallel assay of excavated gates was performed, and in each of the gates used, there were three wells. A collagen sponge that was soaked with BMP-2 was placed in the left end well. At the opposite end, another collagen sponge was placed to which Amniomax medium was added. Once both sponges were soaked, 50,000 cells were seeded in the central well.

In this assay, the study groups were: Fibroblasts (*n* = 8) and MSCat (*n* = 8).

In each of the gates, there were BMP-2 and control (no BMP-2). The times at which samples were collected were: 24 h; 48 h; 7 d; and 14 d, at a rate of two samples per time and study group.

### 2.5. Analysis of Titanium Surface Behavior

- Enzymatic Treatment

Once the cells (fibroblasts or MSCat) had reached semi-confluence (80% of the coated culture flask), enzymatic treatment with a trypsin solution was carried out for 5 min at 37 °C. For this purpose, a trypsin solution was prepared in HBSS buffer free of calcium and magnesium. Once the enzyme solution was prepared, the culture medium was removed using a 5 mL pipette. The cells were washed with a suspension of 1% HBSS in sterile distilled water to remove any remaining culture medium, and for the removal of calcium or magnesium, which may influence the adhesion of the cells to the culture surface. One percent trypsin solution, that was previously prepared in HBSS, was added and incubated at 37 °C for 5 min, and sufficient time was given for the cells to detach from their substrate. The control of the incubation time was vital, as trypsin has a potential deleterious effect on cells, wherein a longer exposure time can result in the death of cells by cytotoxicity.

Following 5 min of incubation, 10 mL of culture medium with 20% fetal bovine serum was added. The proteins present in the serum block the enzyme, and it remains active, thereby blocking the enzymatic action on the cells in the culture medium. After deactivation of the enzyme, the cell suspension was collected in a conical tube, and centrifuged at 1050 rpm for 10 min. The supernatant was removed, and the cell pellet was re-suspended in 1 mL of Amniomax medium. Further to that, the cells were suspended at a rate of 5 × 10^5^ cells/mL.

- Planting and Study Times. For this part of the procedure, the three study groups were used for each cell lineage (Fibroblasts and MSCat cells):
  ○ Control group (untreated screw);
  ○ Screws treated with carboxyethylphosphonic acid;
  ○ Screws treated with carboxyethylphosphonic acid plus BMP-2.

The same seeding methodology was utilized for all the three study groups. Firstly, the screws were placed in a 96-well plate. Each screw was seeded with about 5 × 10^4^ viable cells in 100 µL Amniomax medium. The plate was then incubated at 37 °C for 30 min in an incubator at 5% CO and 99% humidity. Under these conditions, cell adhesion on the different seeded surfaces was ensured. Following which, 200 µL of Amniomax medium was added to each of the wells, and incubated under the same conditions described above for the different study times (24 h, 3, 7,1 4, 21, and 28 days). The culture medium was renewed every 24 h. The number of samples for each cell lineage and each study group was 4 at each time interval. Each cell lineage was seeded in different plates to avoid possible contamination. The rest of the wells in each of the plates not occupied by the trunks belonging to each group were used as a control of cell growth at each time interval.

- SEM observation. At each study time interval, four samples were obtained for each cell lineage and study group. Each sample was fixed for 2–4 h in 0.3% glutaraldehyde solution. After the fixation time, the glutaraldehyde residues were removed by immersing samples in a Millionig buffer solution. After the washing process, the samples were processed for observation under the scanning electron microscope. Once fixed, the pieces were dehydrated using increasing series of acetone (30, 50, 70, 90 and 100%) for 10 min per series. Subsequently, the dehydrated parts were mounted in filter paper cases for subjecting them to a critical point in a Polaron E-3000 with CO2. Once completely dried, the pieces were mounted on steel plates (ANAME), and metallized with gold-palladium in a POLARON metallizer. The samples were processed in this manner for observation by SEM on a DSM-950 microscope (Carl-Zeiss). The state of the cells on the surface of the screws belonging to each study group at different time intervals were observed using SEM.

### 2.6. Identification and Phenotypic Characterization

#### 2.6.1. Immunohistochemical Tests

- Conventional immunohistochemistry. The technique used to detect the antigen-antibody reaction was avidin-biotin labeled with alkaline phosphatase. The labeling was studied with an optical microscope (ZEISS, Jena, Germany), and the same biological material to which the primary antibody had not been added was used as a negative control, being replaced by washing buffer (PBS).
- Immunofluorescence. The marking was performed on tissue fixed with 10 % formaldehyde. Kerosene embedding, microtomy, and deparaffinization of the sections were performed according to the technique used for optical microscopy. The antigen–antibody reaction was detected using a reaction amplification system (TSA TM Plus Fluorescence Systems; Perkin Elmer Life Sciences, Boston, MA, USA), with fluorescein isothiocyanate (FITC) or cyanine 3 (Cy 3). The same biological material to which the primary antibody had not been added was used as a negative control, and was replaced by a buffer solution.

#### 2.6.2. Enzyme-Linked Immunosorbent Assay (ELISA)

MSCat and Fibroblast cells were plated into 96-well plates at a density of 5 × 10^4^ cells/well divided and 200 µL Amniomax medium with 100 ng/mL BMP-2 (Sigma Aldrich San Luis, MO, USA). Cells cultured in the same Amniomax medium without BMP-2 were considered as the control group. The culture media for each group was collected at T0 (immediately after plating), 30 min, 1 h, 2 h, and 24 h after plating, and kept at −80 °C until use.

The levels of BMP-2 secreted by the cells (fibroblasts and MSCat cells) were determined by an enzyme-linked immunosorbent assay (ELISA) technique using a specific kit for this protein (ABCAM). The technique involved a sandwich-type enzyme immunoassay for the quantitative determination of BMP-2 levels released into the culture medium from both fibroblasts and MSCat. The kit was provided with a microplate pretreated with a specific antibody against rat BMP-2. The principle of the technique was in the realization of a standard curve for which the kit was provided with a sample of BMP-2 of known concentration (2000 pg).

A stock solution of BMP-2 at a concentration of 1000 pg/mL was prepared for performing the standard curve. From this dilution, successive dilutions were performed. In this method, different standard samples were obtained at different known concentrations, and each dilution contained 50% of the protein of the immediately preceding dilution, resulting in a gradient of dilutions between 1000–15.6 pg/mL. The blank was performed using the culture medium in order to remove the noise from the medium. At the time of performing the test, the samples of the medium from the fibroblast and MSCat cultures were thawed and centrifuged at 1000 rpm for the removal of cellular debris that may remain, and the supernatant was used to perform the measurements. On the wells of the labeled plate from the kit, 100 µL of each standard solution was added to it.

This process was carried out in duplicate, and fourteen wells (seven dilutions) were used, and two other wells were used to make the blank. The rest of the wells were used for the determination of the BMP-2 levels at the different times of study and for the different cell types by the addition of 100 µL of each test solution for each well, performing the tests in duplicate. Once the entire plate was covered, it was placed in incubation for two hours at 37 °C. Following which, the content of each well was removed, and each well was washed with 400 µL of washing solution from the commercial kit, prepared according to the manufacturer’s instructions. This solution was then removed, and the reagent A solution was added according to the manufacturer’s instructions. It was incubated for one hour at 37 °C, and, subsequently, it was washed again with the washing solution for 1–2 min. The washing solution was then removed, and this process was repeated for three cycles. After this process, 100 µL of working solution B (according to the manufacturer’s instructions) was added, and the plate was incubated for 30 min at 37 °C. Following incubation, the solution was removed, and the wells were washed as described above. Finally, 90 L per well of the chromogenic substrate solution (TMB) was added and incubated for 15–20 min at 37 °C until the color of the wells turned blue.

#### 2.6.3. Molecular Studies (qRT-PCR)

With q-PCR, the amount of BMP-2 mRNA was quantified in both fibroblast and MSCat cell cultures.

RNA extraction was performed by the guanidine isothiocyanate-phenol-chloroform method of Chomczynski and Sacchi [21].

The integrity of the extracted RNA was checked through a 1% agarose gel containing 7 µL of SYBR Green II RNA gel stain (10,000× *g* concentrate in DMSO) (Invitrogen, Carlsbad, CA, USA). 2 µL of RNA was mixed with 8 µL of RNAse-free water and 2 µL of BlueJuice™ Gel Loading Buffer (Invitrogen, Carlsbad, CA, USA) containing bromophenol blue. Electrophoresis was carried out at 80 V for about 50 min. Observation of the bands was performed with the aid of a transilluminator, and samples showing RNA degradation were discarded.

The concentration of each sample was quantified by measuring the absorbance at 260 nm in an Ultrospec 3100 Pro spectrophotometer (Amersham Biosciences, Piscataway, NJ, USA). Once the concentration of each sample was known, all were brought to the same concentration of 50 ng/µL.

Using reverse transcription, cDNA was synthesized from 500 ng of total RNA. For this purpose, 10 µL of RNA (50 ng/µL) was mixed with 10 µL of 2X RT mix prepared from the kit components (High Capacity cDNA Reverse Transcription, Applied Biosystems, Foster City, CA, USA).

In the first step, the reaction was subjected to an RNA denaturation cycle at 25 °C for 10 min in a thermal cycler. Subsequently, it was subjected to a cDNA synthesis cycle at 37 °C for 2 h. Finally, an enzyme denaturation cycle was performed at 85 °C for 5 s. The cDNA obtained during the reverse transcription process was diluted at a ratio of 1:20, and stored at −80 °C.

- In vitro retrotranscription. Retrotranscription (RT) reactions of the messenger RNAs were performed to give rise to the corresponding cDNAs using the kit.
- Real-time PCR. Roche’s commercial Lightcycler FastStart DNA Master SYBR GreenI kit was used to perform the real-time PCR reactions. Once the reaction mixture was generated, it was subjected to a program of PCR cycles performed in the Roche Lightcycler thermocycler.

The fluorescence emitted by the SYBGreen I probe during the real-time PCR reactions described above was analyzed using LightCycler 4.05 software (Roche, Basel, Switzerland), and crossing point (CP) values were obtained for the target gene and the reference gene (GAPDH) in each of the samples. A calibration curve was included in each real-time PCR reaction, and the reaction efficiency value was calculated from its slope. For each sample, the amounts corresponding to the target gene and the reference gene were determined by interpolation with the standard curve. Subsequently, the DNA content (percentage) was calculated as the ratio between the amount of the target gene sequence and the amount of the reference gene sequence.

To check for the presence of a single PCR product, a 2% agarose gel (SeaKem GTG Agarose, Cambrex, Rockland, ME, USA) was run in 1× TBE buffer, to which 7 L of SYBR Green was added (Table 1).

### 2.7. Statistical Analysis

Statistical tests were performed using the SPSS Macros software. The F-test was performed for analysis of variances to assess the surface wettability. As there were unequal variances, a modification of the t-test, known as the Welch test, was performed. Values of *p* < 0.05 were considered as statistically significant.

## 3. Results

### 3.1. Wettability

**Figure 4:**
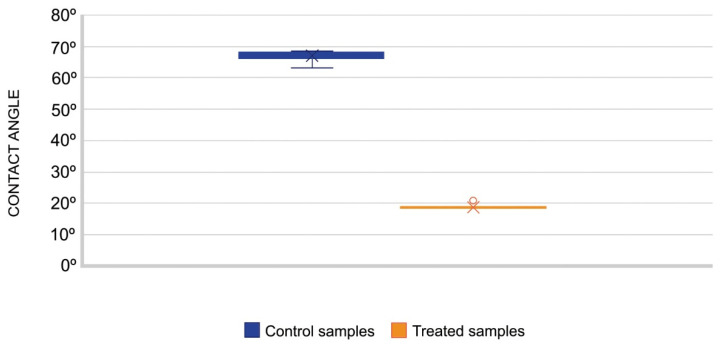
Boxplots showing the contact angle obtained for the control samples (blue) with its median (67.8°), minimum (63.2°), and maximum (68.6°); and the treated samples (orange) with its median (18.65°), minimum (18.3°), and maximum (20.8°).

The results of the F-test showed that there were unequal variances between the two sample groups, and the Welch test observed the mean contact angle to be 67 for the control group, and 18.84 for the test group. This leads to a value of t = 77.06 to relate to a Student’s t distribution with approximately 12 degrees of freedom. For α = 0.05, the reference critical value of 1.78 indicates that there were statistically significant differences in the contact angle measurements between the control group and the test group treated with carboxyethylphosphonic acid. The box plots for the variation of the contact angles in each group has been depicted in Figure 4.

### 3.2. Surface Texture Analysis Using SEM

**Figure 5:**
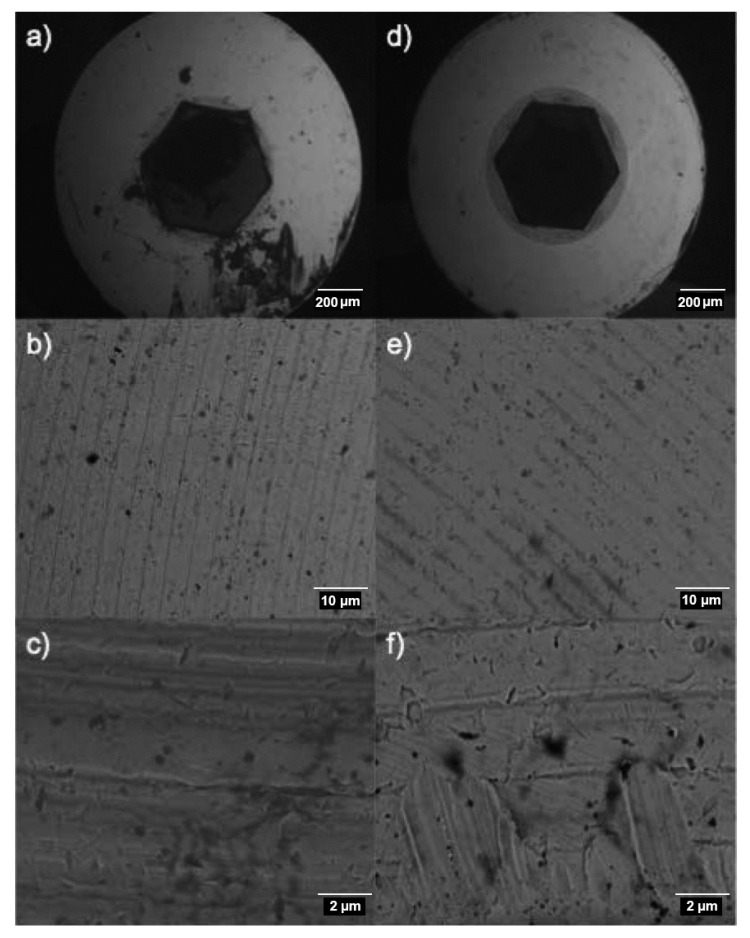
SEM. study of the cranial surface of the screws belonging to the control group (**a**–**c**) and treated with carboxylphosphonic acid (**d**–**f**); (**a**,**d**) 50×; (**b**,**e**) 1000×; (**c**,**f**) 3000×.

It was observed that the cranial side of the screw surface did not differ from the control group at lower magnification, but at higher magnifications, the surface roughness increased in the Ti screws treated with carboxylphosphonic acid. The increase in texture was evident in higher magnifications of 3000×. At the micrometer scale, granules appeared as ‘peak and valley’ type surface structures due to the erosive process during the fabrication of the substrate, with the acid-treated group having a more complex surface compared to the control group. At higher magnifications of 1000× and 3000×, a greater number of cracks and ‘pecked’ images appeared on the surface of the acid-treated logs, indicative of a higher surface texture, mainly due to the corrosive action of the acid that causes small structural modifications on the surface of the screws, which became more evident at higher magnifications (3000×) (Figure 5).

### 3.3. Protein Immobilization

**Figure 6:**
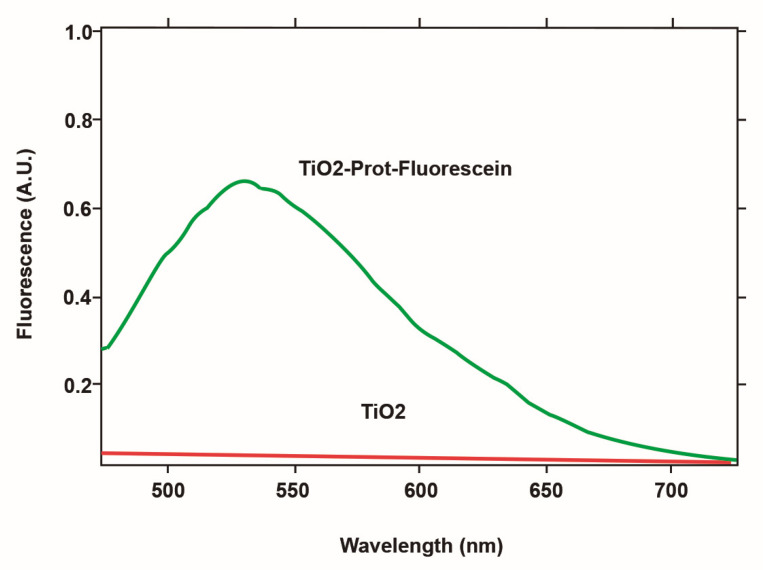
Fluorescence emission spectrum of the protein monolayer on a TiO_2_ surface—the red line indicates the absence of fluorescence when there is no protein on the surface.

The immobilization of proteins on the control and test groups of screws was assessed using glucose oxidase, and analyzed with NHS-Fluorescein tagging. A fluorescence was observed with titanium at a wavelength of 533 nm, confirming the presence of immobilized protein on the surface of the titanium using this method. The addition of fluorescein to a phosphonic acid-treated screw generated a surface that did not show fluorescence, and it can be concluded that this process is viable, and that the fluorescence seen in the Ti could be due to the immobilization of the protein (Figure 6).

### 3.4. Cellular Behavior—Fibroblasts

Proliferation studies showed that fibroblasts presented a characteristic spindle-shaped morphology at 24 h. A high proliferation index, typical of these cells in culture, was observed, resulting in the observation of numerous mitosis figures in the fibroblast cultures at this time. Incubation of fibroblasts with BMP-2 did not affect the morphology of these cells, which remained spindle-shaped. In comparison with fibroblasts from control cultures, an increase in cell proliferation could be observed from the initial moments of the culture.

Migration studies showed that BMP-2 also induced a higher rate of cell migration, demonstrating the high chemotactic power of this molecule. Observation of the excavated wells revealed an increased cell migration from the central well to the well where it had been incubated with BMP-2. This translated into the presence of a greater number of cells in the wells treated with BMP-2 from the initial moments of the culture, and was maintained until 24 h after the start of the culture, at which time, cells began to be seen in the untreated well. The use of BMP-2 in the treated wells did not induce morphological differences, but the presence of a greater number of dividing cells as a consequence of the induction with BMP-2 was easily observed.

**Figure 7:**
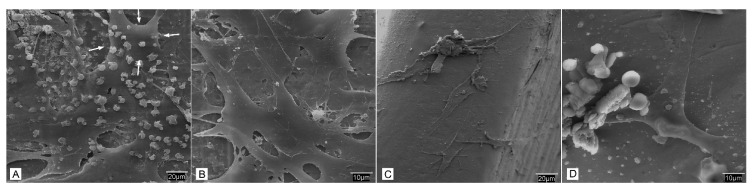
SEM Study of the sowing of fibroblasts in different incubation periods: (**A**) Control screws after 24 h at 500× in which zones are observed where the adhered cells present a more polygonal morphology with many vesicles on their surface (arrows); (**B**) Control screws after 24 h at 1000×; (**C**) Control screws after 28 days at 500×; (**D**) Control screws after 28 days at 1000× in which a large amount of cellular detritus is observed.

In relation to cell behavior on the titanium surface, the cellular behavior the fibroblasts was studied following implantation on the titanium surfaces, either in the control group or those treated with carboxyethylphosphonic acid. After 24 h, microscopic examination revealed a smooth surface, but there was no homogenous adhesion on the titanium screws in the control group. The fibroblasts adhered to the screws had a more polygonal morphology with numerous vesicles on the surface. There is progressive dislocation of the seeded fibroblasts from the titanium surface at around 14–21 days, and there is a vast amount of cellular debris observed on the surface (Figure 7).

**Figure 8:**
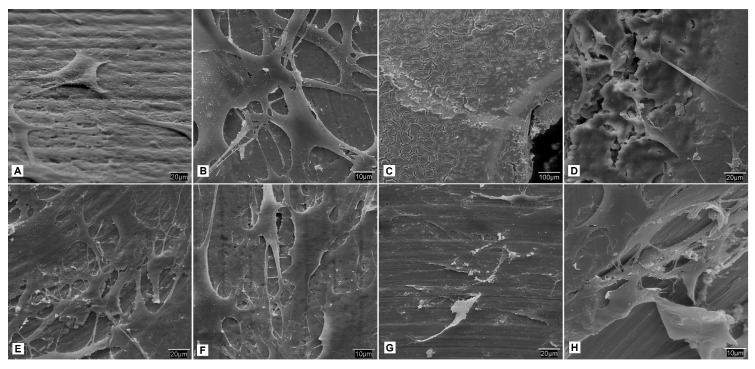
S.E.M. Study of the sowing of fibroblasts in different incubation periods: (**A**) Acid + BMP-2 treated screws after 24 h at 500×; (**B**) Screws treated with acid + BMP-2 after 24 h at 1000× where it can be seen how the cells adhere and expand over the treated surface of the titanium, growing in multilayer, which contrasts with the large amount of vesicles observed previously in the control group; (**C**) Screws treated with acid + BMP-2 after 14 days at 100×; (**D**) Screws treated with acid + BMP-2 after 14 days at 500×; (**E**) Screws treated with acid + BMP-2 after 21 days at 500×; (**F**) Screws treated with acid + BMP-2 after 21 days at 1000×; (**G**) Screws treated with acid + BMP-2 after 28 days at 500×; (**H**) Screws treated with acid + BMP-2 after 2 at 1000× where areas of slight cellular denudation are evident.

A contrasting finding was observed with fibroblasts seeded on the test groups treated with the acid and BMP-2, where there was increased surface roughness on the screws. At higher magnifications, the fibroblast cells exhibited a smooth surface with good adhesion and expansion on the Ti surface at 24 h. Following 7 to 21 days of seeding, the cells formed several layers with greater homogeneity across the Ti surface (Figure 8).

### 3.5. Cellular Behavior—MSCat Cells

Proliferation studies showed that MSCat cells in culture presented a polygonal morphology with a spherical and centered nucleus, characterized by the presence of several nucleoli, demonstrating a high activity of these cells in culture. The characteristics of these cells as undifferentiated cells are evidenced by their high proliferative and self-renewal power.

BMP-2 treatment of mesenchymal cells from adipose tissue maintained a polygonal morphology, similar to that described for the cultures of these cells in the groups that were not treated with this bone morphogenetic protein. In this group, it is worth noting a decrease in the proliferative power of these cells, caused fundamentally by the influence of this protein on the possible differentiation of these mesenchymal cells.

In the migration study, it was demonstrated, as in the fibroblasts, that BMP-2 also induced a higher rate of cell migration of MSCat cells.

**Figure 9:**
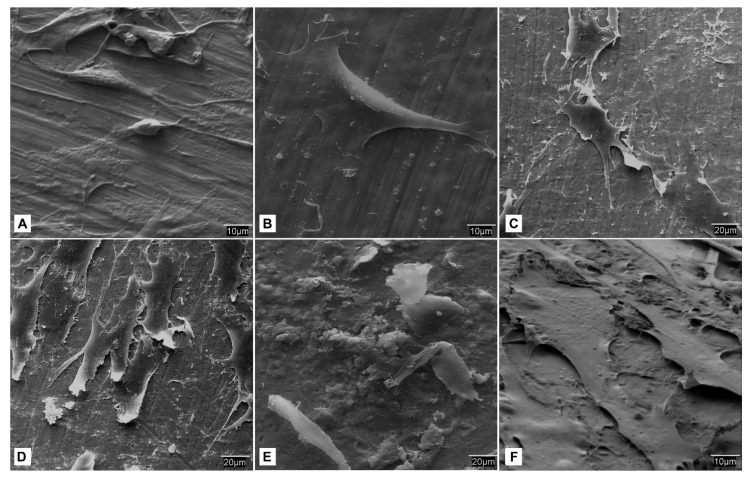
SEM Study of the sowing of MSCat in different incubation periods: (**A**) shows seeding on control screw observed at 1000× after 24 h; (**B**) shows seeding on control screw observed at 1000× after 7 days; (**C**,**D**) shows seeding on control screw observed at 500× after 14 days; (**E**) shows seeding on control screw observed at 500× after 21 days; (**F**) at 1000× from the surface of a screw belonging to the control group after 28 days of incubation where the formation of a multilayer with a slight dislocation of the most superficial cellular layer is observed.

A similar process of seeding of mesenchymal stem cells was performed on the Ti screws in the control group and the test group treated with carboxyethylphosphonic acid and BMP-2. After 24 h, there was uniform coating of mesenchymal cells on the Ti surface in the control group, and the cells appeared with varying morphologies from fusiform, flattened, to polygonal shapes. After 21 days, there was instability of the cellular layer, and cellular denudation was observed (Figure 9).

**Figure 10:**
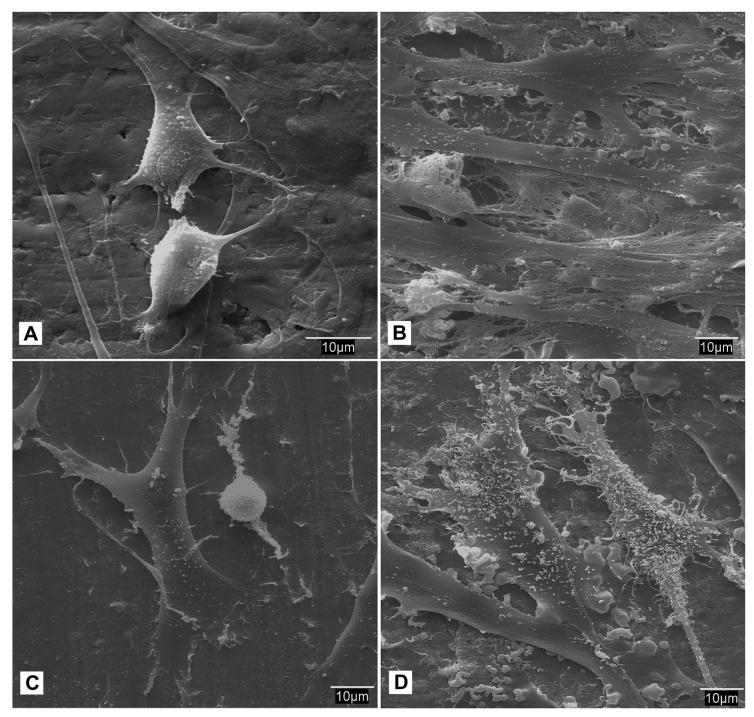
SEM Study of the sowing of MSCat in different incubation periods: (**A**) screws treated with BMP-2 and incubated with MSCat after 24 h at 1000×, the presence of dividing cells can be observed; (**B**) screw treated with BMP-2 after 7 days of culture with MSCat where deposits on the surface of a large quantity of secretion products can be seen at 1000×; (**C**) screw treated with BMP-2 after 14 days of culture with MSCat at 1000×; (**D**) screws treated with BMP-2 after 28 days of seeding at 1000×.

The MSCat cells seeded on the test specimens had a more stable layer, with cells having a fusiform morphology and stable anchorage on the surface of the titanium. Around 14 and 21 days of observation, there were several layers of cells with high stability, and the superficial layers had a pronounced secretory activity suggestive of a higher proliferation (Figure 10).

### 3.6. Identification and Phenotypic Characterization

#### 3.6.1. Immunohistochemical Tests

**Figure 11:**
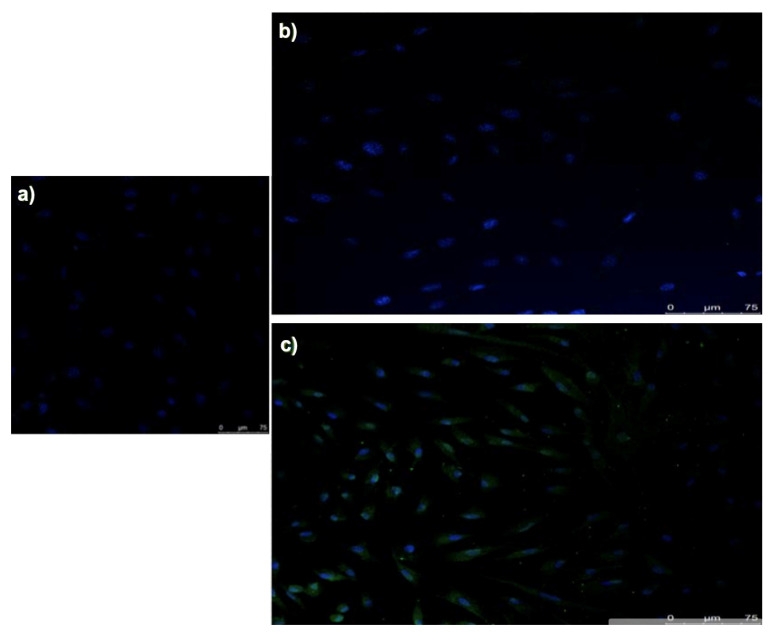
BMPR-II determination by immunofluorescence techniques: (**a**) negative control; (**b**) fibroblast cultures; and (**c**) MSCat cultures.

Regarding immunofluorescence techniques for the type II BMP receptor, a very homogeneous distribution was observed along the membrane of the expanded fibroblasts in culture, affecting approximately 75% of the cells. As for MScat cells, a very pronounced labeling was observed on the membrane of isolated cells, which could demonstrate a higher degree of differentiation of some of these cells (Figure 11).

#### 3.6.2. Enzyme-Linked Immunosorbent Assay (ELISA)

**Figure 12:**
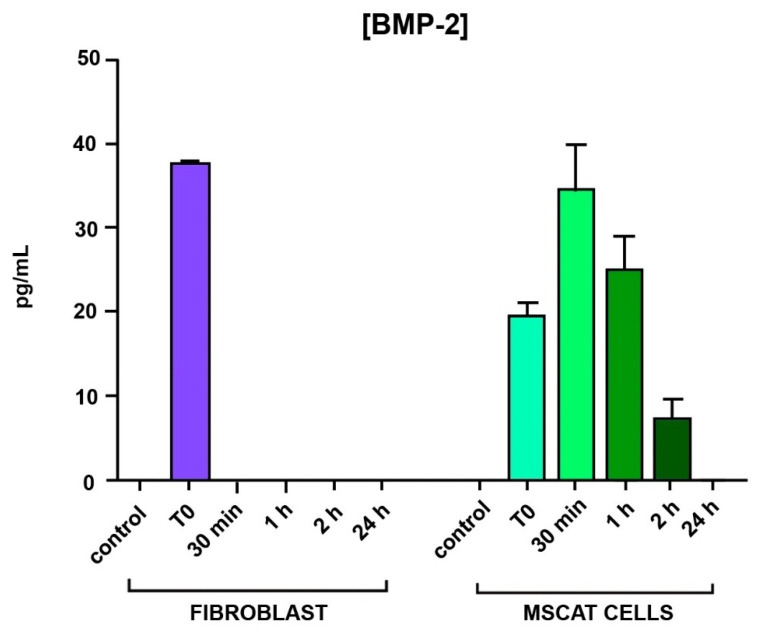
Measurements of BMP-2 levels in fibroblast and MSCat cultures by enzyme-linked immunoassay (ELISA) techniques at different study times.

No BMP-2 release to the medium was detected in either fibroblast or MSCat control cultures at any time. The addition of BMP-2 to the culture media caused a different behavior between fibroblasts and MSCat cells: 30 min after treatment, no presence of BMP-2 was observed in the fibroblast cultures. On the other hand, 30 min after stimulation, a progressive increase in BMP-2 levels in the MSCat cell cultures was observed. Significant differences were observed between 30 min and two hours after the stimulus between both cell lines. In the absence of a new stimulus pulse, the secretion of BMP-2 by these cells decreases over time, disappearing completely 24 h after the initial stimulus (Figure 12).

#### 3.6.3. Molecular Studies

**Figure 13:**
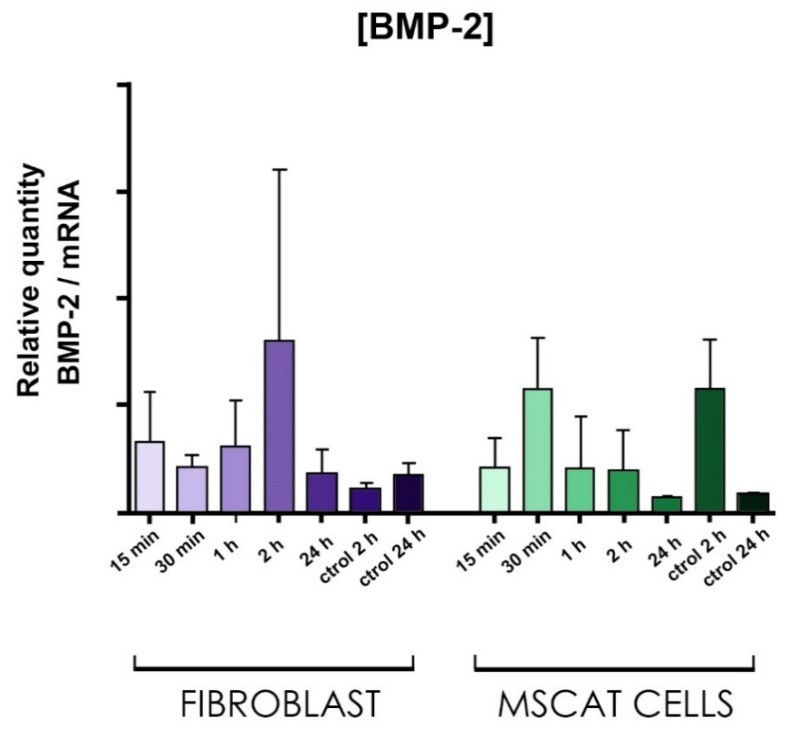
Graph showing the determination of messenger RNA levels for BMP-2 in fibroblast and MSCat cultures measured by q-PCR-RT at different study times: 15 min; 30 min; 1 h; and 2 h.

MSCat and fibroblast cultures were used to undergo osteogenic differentiation induced by bone morphogenetic protein type II (BMP-2) treatment, and the expression of messenger RNA levels for BMP-2 was quantified at different study times. The results of the expression of messenger RNA specific for BMP-2 can be seen in Figure 13.

## 4. Discussion

There has been a tremendous increase in the research activity towards improving the biological activity on the Ti and Ti alloy surfaces for the promotion of processes such as osseointegration and soft tissue healing [22]. The conventional methods of surface modification involved a top down approach involving multiple processes and special guidance, whereas the emergence of nanotechnology has given rise to self-assembly, which is a bottom up process that does not require specific guidance or an intervention during the process of assembly [23].

In this in vitro study, it was observed that when there was an increase in surface energy, it gets translated to higher surface wettability to blood, improved cellular adhesion, and increased fibrin binding, matrix proteins, and differentiation factors. This can influence cellular behavior such as proliferation, adhesion, and migration, and thereby promote osseointegration and tissue healing following dental implant placement [24]. A study conducted by Lan et al. [25] observed the water contact angles and the surface wettability characteristics of a TiO_2_-modified surface with four types of phosphonic acid compounds, along with the control. It was found that the TiO_2_ surface treated with 11-phosphonoundecanoic acid (PUA) had the best hydrophilic surface, with an average contact angle measurement of 68.8 ± 0.7. The wettability and contact angle measurement were based on the premise that when a material is placed in a biological surrounding, there is adsorption of water to the material’s surface in the first few nanoseconds. Thus, wettability of the material can determine its biological interactions, and, thus, moderate hydrophilicity can favor cellular adhesion [26]. In this study, the average contact angle for the control group was 67, and was 18.84 for the test group.

The SEM analysis of the Ti screws in both the groups revealed that there was a considerable increase in the surface texture in the test group treated with the acid. The appearance ranged from ‘peaks and valleys’ to a more cracked appearance on the Ti surface. The surface texture and roughness on an implant can affect the rate at which osseointegration occurs, and biomechanical fixation of the titanium to the bone [27]. The surface roughness has been observed to play a significant role in protein adsorption and adhesion of osteoblasts, which, in turn, can have an influence on the process of osseointegration [28]. The increased surface texture observed in the test group in this study could play an important role in the cellular behavior, as well as vital processes, such as primary stability and osseointegration.

In this study, the immobilization of proteins revealed that there was significant fluorescence of the Ti surface observed at 533 nm, suggestive of immobilized proteins. Similar research, conducted by Tack et al. [29], observed an increase in immobilized proteins using a similar method to this study, and the carboxyl functionalized group had the highest protein, followed by the hydroxyl group. In this study, it was observed that cellular behavior such as cell proliferation, migration, and morphology was improved on the treated surfaces with acid and BMP-2 when compared to the control group. A study by Viornery et al. [30] observed Ti discs following incubation in ethane-1,1,2-triphosphonic acid (ETP), and showed that there was increased differentiation, proliferation, and production of proteins when rat osteoblastic cells (CRP10/30) were seeded onto the functionalized surface.

The cellular behavior of fibroblasts and MSCat cells were observed in this study following culture and seeding of these cell lines on the different study groups of Ti screws. The fibroblasts had a more polygonal shape with numerous vesicles in the experimental group with acid-treated Ti screws than the control group. There was also limited cellular adhesion in the control group. The study group treated with acid and BMP-2 also showed fibroblasts with a smoother surface and increased expansion, along with adhesion. Similar findings were observed with the MSCat cell line on the experimental groups, and there was a uniform coating of it. Mani et al. observed that SAMS on Ti surfaces prepared from an aqueous solution of flufenamic acid via esterification reported the interaction of these SAMs with human aortic endothelial cells (HAECs). It was shown that the cellular adhesion was the highest in SAMs when compared to plain glass or the control Ti surface, although the rate of proliferation was slower. The cells also showed a polygonal morphology [31]. Although a direct comparison cannot be made with this study, it is reasonable to assume that functionalization with SAMs can result in a favorable Ti surface for cellular adhesion, and it is worth exploring the mechanism in future research.

This study has assessed the effect of SAMs on Ti surfaces using carboxyethylphosphonic acid. The limitation with the SAMs technique lies in its reproducibility and the production of an ideal surface. The findings from this study can be taken as a preliminary insight into the promising findings with the usage of this nanotechnology in performing surface modification on Ti surfaces for improving osseointegration and wound healing. Further studies using an Alizarin Red S staining assay can be performed to assess the cellular behavior at a further level. Even though the results at the laboratory stage show promise, the clinical implications of the utilization of this technique, clinical application for dental implantology, and the shelf-life of these functionalized implant surfaces remain to be explored.

## 5. Conclusions

In this in vitro study, it was observed that the treated Ti screws with carboxyethylphosphonic acid had the lowest contact angle for surface wettability. It also showed increased surface roughness when observed under SEM, and an increase in immobilized proteins on its surface. It was also noted that the cellular behavior of fibroblasts and MSCat cells were improved in the test group treated with the acid. The SAM surface modification technique is a promising breakthrough in the field of implantology, but it has to be explored further on its clinical implications and commercial viability.

## Figures and Tables

**Table 1:** Primer used in RT-PCR.

Gene	S Primer Sequence (5′→3′)	AS Primer Sequence (5′→3′)	Banding Temperature
BMP-2	CCA GGT TAG TGA CTC AGA ACA C	TCA TCT TGG TGC AAA GAC CTG C	60°

## References

[1] Pjetursson B.E., Heimisdottir K. (2018). Dental implants—Are they better than natural teeth?. Eur. J. Oral. Sci..

[2] Branemark P. (1983). Osseointegration and its experimental background. J. Prosthet. Dent..

[3] Jemat A., Ghazali M.J., Razali M., Otsuka Y. (2015). Surface Modifications and Their Effects on Titanium Dental Implants. BioMed Res. Int..

[4] Barros R.R.M., Novaes A.B., Papalexiou V., Souza S.L.S., Taba M., Palioto D.B., Grisi M.F. (2009). Effect of biofunctionalized implant surface on osseointegration: A histomorphometric study in dogs. Braz. Dent. J..

[5] Beutner R., Michael J., Schwenzer B., Scharnweber D. (2010). Biological nano-functionalization of titanium-based biomaterial surfaces: A flexible toolbox. J. R. Soc. Interface.

[6] Lutz R., Srour S., Nonhoff J., Weisel T., Damien C.J., Schlegel K.A. (2010). Biofunctionalization of titanium implants with a biomimetic active peptide (P-15) promotes early osseointegration: Biofunctionalization of titanium implants with a biomimetic active peptide. Clin. Oral. Implant. Res..

[7] Hanawa T. (2010). Biofunctionalization of titanium for dental implant. Jpn. Dent. Sci. Rev..

[8] Tan G., Zhang L., Ning C., Liu X., Liao J. (2011). Preparation and Characterization of APTES Films on Modification Titanium by SAMs. Thin Solid Films.

[9] Spori D.M., Venkatarman N.V., Tosatti S.G.P., Durmaz F., Spencer N.D., Zürcher S. (2007). Influence of Alkyl Chain Length on Phosphate Self-Assembled Monolayers. Langmuir.

[10] Mann J.R., Nevins J.S., Soja G.R., Wells D.D., Levy S.C., Marsh D.A., Watson D.F. (2009). Influence of Solvation and the Structure of Adsorbates on the Kinetics and Mechanism of Dimerization- Induced Compositional Changes of Mixed Monolayers on TiO_2_. Langmuir.

[11] Ajami E., Aguey-Zinsou K.F. (2011). Formation of OTS Self- Assembled Monolayers at Chemically Treated Titanium Surfaces. J. Mater. Sci. Mater. Med..

[12] Bozzini S., Petrini P., Tanzi M.C., Zürcher S., Tosatti S. (2010). Poly(ethylene glycol) and Hydroxy Functionalized Alkane Phosphate Mixed Self-Assembled Monolayers to Control Nonspecific Adsorption of Proteins on Titanium Oxide Surfaces. Langmuir.

[13] Ulman A. (1996). Formation and Structure of Self-Assembled Monolayers. Chem. Rev..

[14] Love J.C., Estroff L.A., Kriebel J.K., Nuzzo R.G., Whitesides G.M. (2005). Self-Assembled Monolayers of Thiolates on Metals as a Form of Nanotechnology. Chem. Rev..

[15] Liu Q., Ding J., Mante F.K., Wunder S.L., Baran G.R. (2002). The role of surface functional groups in calcium phosphate nucleation on titanium foil: A self-assembled monolayer technique. Biomaterials.

[16] Liu D.P., Majewski P., O’Neill B.K., Ngothai Y., Colby C.B. (2006). The optimal SAM surface functional group for producing a biomimetic HA coating on Ti. J. Biomed. Mater. Res. A.

[17] Shalabi M.M., Wolke J.G.C., de Ruijter A.J.E., Jansen J.A. (2007). Histological evaluation of oral implants inserted with different surgical techniques into the trabecular bone of goats. Clin. Oral. Implant. Res..

[18] Svensson S., Suska F., Emanuelsson L., Palmquist A., Norlindh B., Trobos M., Bäckros H., Persson L., Rydja G., Ohrlander M. (2013). Osseointegration of titanium with an antimicrobial nanostructured noble metal coating. Nanomedicine.

[19] Hulander M., Hong J., Andersson M., Gerven F., Ohrlander M., Tengvall P., Elwing H. (2009). Blood interactions with noble metals: Coagulation and immune complement activation. ACS Appl. Mater Interfaces.

[20] Hulander M., Lundgren A., Berglin M., Ohrlander M., Lausmaa J., Elwing H. (2011). Immune complement activation is attenuated by surface nanotopo-graphy. Int. J. Nanomed..

[21] Chomczynski P., Sacchi N. (1987). Single-step method of RNA isolation by acid guanidinium thiocyanate-phenol-chloroform extraction. Anal. Biochem..

[22] Shi Q., Qian Z., Liu D., Liu H. (2017). Surface Modification of Dental Titanium Implant by Layer-by-Layer Electrostatic Self-Assembly. Front. Physiol..

[23] Kumar S.M., Balakrishnan P.K., Hedge C., Dandekeri S. (2020). Self-Assembled Monolayer- A Nano Surface Modification. J. Evol. Med. Dent. Sci..

[24] Anil S., Anand P.S., Alghamdi H., Janse J.A. (2011). Dental Implant Surface Enhancement and Osseointegration. Implant. Dent. A Rapidly Evol. Pract..

[25] Lan W.C., Huang T.S., Cho Y.C., Huang Y.T., Walinski C.J., Chiang P.C., Rusilin M., Pai F.T., Huang C.C., Huang M.S. (2020). The Potential of a Nanostructured Titanium Oxide Layer with Self-Assembled Monolayers for Biomedical Applications: Surface Properties and Biomechanical Behaviors. Appl. Sci..

[26] Chen R.S., Chen Y.J., Chen M.H., Young T.H. (2007). Cell-surface interactions of rat tooth germ cells on various biomaterials. J. Biomed. Mater. Res. A.

[27] Boyan B.D., Lohmann C.H., Dean D.D., Sylvia V.L., Cochran D.L., Schwartz Z. (2001). Mechanisms Involved in Osteoblast Response to Implant Surface Morphology. Annu. Rev. Mater. Res..

[28] Brett P.M., Harle J., Salih V., Mihoc R., Olsen I., Jones F.H., Tonetti M. (2004). Roughness response genes in osteoblasts. Bone.

[29] Tack L., Schickle K., Böke F., Fischer H. (2015). Immobilization of specific proteins to titanium surface using self-assembled monolayer technique. Dent. Mater..

[30] Viornery C., Chevolot Y., Léonard D., Aronsson B.-O., Péchy P., Mathieu H.J., Descouts P., Graetzel M. (2002). Surface Modification of Titanium with Phosphonic Acid to Improve Bone Bonding: Characterization by XPS and ToF-SIMS. Langmuir.

[31] Mani G., Chandrasekar B., Feldman M.D., Patel D., Agrawal C.M. (2009). Interaction of endothelial cells with self-assembled monolayers for potential use in drug-eluting coronary stents. J. Biomed. Mater. Res. B Appl. Biomater..

